# Challenges in the Implementation of the NeoOBS Study, a Global Pragmatic Observational Cohort Study, to Investigate the Aetiology and Management of Neonatal Sepsis in the Hospital Setting

**DOI:** 10.3390/antibiotics12050923

**Published:** 2023-05-17

**Authors:** Amy Riddell, Aislinn Cook, Nathalie Khavessian, Sally Ellis, Davide Bilardi, Erika Correia, Tomislav Kostyanev, Alessandra Nardone, Neal Russell, Tuba Vilken, Wolfgang Stohr, Bethou Adhisivam, Iana Rosa Alves de Moraes, Nawshad Uddin Ahmed, Adrie Bekker, Eitan Naaman Berezin, Suppawat Boonkasidecha, Cristina G. Carvalheiro, Prachi Chauhan, Sara Chiurchiù, Elisavet Chorafa, Angela Dramowski, Madhusudhan DS, Jinxing Feng, Shengnan Jia, Yuan Kong, Mary Kyohere, Angeliki Kontou, Sorasak Lochindarat, Maia De Luca, Aripfani Mphaphuli, Marisa M. Mussi-Pinhata, Sheila Murunga, Firdose Lambey Nakwa, Sushma Nangia, Erinah Nassolo, Ngoc Thi Bin Hoang, Christina W. Obiero, Linus Olson, Wang Ping, Nishad Plakkal, Priyanka Prasad, Kanchana Preedisripipat, Sheikh Wasik Rahman, Tiffany Seef, Pra-ornsuda Sukrakanchana, Reenu Thomas, Zhang Yu, Qiaoru Zhang, A. Sarah Walker, Julia Bielicki, Paul T. Heath, Michael Sharland, Tatiana Munera-Huertas

**Affiliations:** 1 Centre for Neonatal and Paediatric Infection, St. George’s University of London, London SW17 0RE, UK; 2 GARDP, 1202 Geneva, Switzerland; 3 Fondazione Penta ETS, 35127 Padova, Italy; 4 Laboratory of Medical Microbiology, Vaccine & Infectious Disease Institute, University of Antwerp, 2610 Antwerpen, Belgium; 5 MRC Clinical Trials Unit, University College London, London WC1V 6LJ, UK; 6 Department of Neonatology, Jawaharlal Institute of Postgraduate Medical Education & Research (JIPMER), Pondicherry 605006, India; 7 Santa Casa de São Paulo, Sao Paulo 01221-010, Brazil; 8 Child Health Research Foundation, Dhaka 1207, Bangladesh; 9 Department of Paediatrics and Child Health, Faculty of Medicine and Health Sciences, Stellenbosch University, Cape Town 8000, South Africa; 10 Queen Sirikit National Institute of Child Health, Bangkok 10400, Thailand; 11 Department of Pediatrics, Ribeirão Preto Medical School, University of São Paulo, Ribeirão Preto 14049-900, Brazil; 12 Department of Neonatology, Lady Hardinge Medical College and Kalawati Saran Children’s Hospital, New Delhi 110001, India; 13 Academic Department of Pediatrics (DPUO), Infectious Disease Unit, Bambino Gesù Children’s Hospital, IRCCS, 00165 Rome, Italy; 14 Infectious Diseases Unit, 3rd Dept Pediatrics, School of Medicine, Faculty of Health Sciences, Aristotle University and Hippokration General Hospital, 546 43 Thessaloniki, Greece; 15 Seth G. S. Medical College & KEM Hospital, Mumbai 400012, India; 16 Department of Neonatology, Shenzhen Children’s Hospital, Shenzhen 518048, China; 17 Department of Neonatology, Beijing Children’s Hospital, Capital Medical University, National Center for Children’s Health, Beijing 100051, China; 18 Clinical Laboratory, Shenzhen Children’s Hospital, Shenzhen 518048, China; 19 MUJHU Research Collaboration, Kampala P.O. Box 23491, Uganda; 20 1st Neonatology Department and Neonatal Intensive Care Unit, School of Medicine, Faculty of Health Sciences, Aristotle University of Thessaloniki, Hippokration General Hospital, 546 42 Thessaloniki, Greece; 21 School of Clinical Medicine, Faculty of Health Sciences, University of Witwatersrand, Johannesburg, South Africa AND Department of Paediatrics and Child Health, Charlotte Maxeke Johannesburg Academic Hospital, Johannesburg 2193, South Africa; 22 Clinical Research Department, KEMRI-Wellcome Trust Research Programme, Kilifi P.O. Box 230, Kenya; 23 Department of Paediatrics and Child Health, School of Clinical Medicine, Faculty of Health Sciences, University of the Witwatersrand, Johannesburg 1864, South Africa; 24 Department of Microbiology, Vietnam National Children’s Hospital, Hanoi 100000, Vietnam; 25 Amsterdam UMC, University of Amsterdam, Emma Children’s Hospital, Department of Global Health, 1081 Amsterdam, The Netherlands; 26 Department of Women’s and Children’s Health, Karolinska institutet, Stockholm, Sweden and Department of Global Public Health, Karolinska Institutet, 171 77 Stockholm, Sweden; 27 Beijing Obstetrics and Gynecology Hospital, Capital Medical University, Beijing 100026, China; 28 Beijing Maternal and Child Health Care Hospital, Beijing 100026, China; 29 Chiangrai Prachanukroh Hospital, Chiang Rai 57000, Thailand; 30 AMS-PHPT Research Collaboration, Chiang Mai University, Chiang Mai 50200, Thailand

**Keywords:** neonatal sepsis, antimicrobial resistance, antibiotic treatment, LMIC, global collaboration, empiric antibiotics, clinical trials

## Abstract

Neonatal sepsis is a significant cause of mortality and morbidity in low- and middle-income countries. To deliver high-quality data studies and inform future trials, it is crucial to understand the challenges encountered when managing global multi-centre research studies and to identify solutions that can feasibly be implemented in these settings. This paper provides an overview of the complexities faced by diverse research teams in different countries and regions, together with actions implemented to achieve pragmatic study management of a large multi-centre observational study of neonatal sepsis. We discuss specific considerations for enrolling sites with different approval processes and varied research experience, structures, and training. Implementing a flexible recruitment strategy and providing ongoing training were necessary to overcome these challenges. We emphasize the attention that must be given to designing the database and monitoring plans. Extensive data collection tools, complex databases, tight timelines, and stringent monitoring arrangements can be problematic and might put the study at risk. Finally, we discuss the complexities added when collecting and shipping isolates and the importance of having a robust central management team and interdisciplinary collaborators able to adapt easily and make swift decisions to deliver the study on time and to target. With pragmatic approaches, appropriate training, and good communication, these challenges can be overcome to deliver high-quality data from a complex study in challenging settings through a collaborative research network.

## 1. Introduction

The high rates of antimicrobial resistance (AMR) to World Health Organization (WHO)-recommended empiric antibiotic regimens for the treatment of neonatal sepsis in low- and middle-income countries (LMICs) [1,2,3,4,5] have led to a significant increase in the use of carbapenems and last-resort treatments such as colistin [6,7,8]. In 2018, an estimated 5.3 million deaths in children <5 years were caused by preventable illnesses, including infections, with almost half of all deaths occurring in the first month of life [9]. The United Nations Sustainable Development Goals (SDGs) aim to reduce global neonatal mortality from 22 per 1000 live births to below 12 per 1000 live births by 2030, which would require substantial decreases in sepsis-related mortality [10,11]. In 2016, the Global Antibiotic Research and Development Partnership (GARDP) was established as a joint initiative by the WHO and Drugs for Neglected Diseases initiative (DNDi). GARDP is working to develop new treatments for antibiotic-resistant infections and collaborates with partners to ensure sustainable access to affordable novel antibiotic treatments and their responsible use to help achieve SDG Goals 3.2 and 3.8: “End preventable deaths of newborns and children under 5 years of age […] (SDG 3.2) and achieve universal health coverage […] and to ensure access to safe, effective, quality, and affordable medicines […] for all” (SDG 3.8) [10].

Several recent studies have reported on the epidemiology of neonatal sepsis in low- and middle-income countries. Three large multi-centre observational cohort studies of neonatal sepsis were recently performed, including the Delhi Neonatal Infection Study (DeNIS) [12], the Burden of Antibiotic Resistance in Neonates from Developing Societies (BARNARDS) [13,14], and in the community setting, the Aetiology of Neonatal Infection in South Asia (ANISA) Study [15]. Despite these contributions, no global prospective observational cohort study of the management and outcomes of sepsis in hospitalised neonates has been undertaken in settings where high rates of antimicrobial resistance are associated with neonatal mortality.

In 2018, the global Neonatal Sepsis Observational Study (NeoOBS) was set up by GARDP, as the sponsor, in collaboration with St George’s, the University of London, the Medical Research Council Clinical Trials Unit at UCL, Laboratory of Medical Microbiology at the University of Antwerp (UA), the Penta Foundation, and a global collaborative network of hospitals, to assess the etiology and management of neonates with significant sepsis (clinicaltrials.gov reference number: NCT03721302). Significant sepsis was defined as presenting with two or more of the signs/symptoms listed in the inclusion and exclusion criteria (Appendix A, Table A1)) [16,17]. This study was a multi-centre, prospective, observational cohort study of inpatient management of neonatal sepsis in 19 sites across 11 countries (Bangladesh, Brazil, China, Greece, India, Italy, Kenya, South Africa, Thailand, Uganda, and Vietnam). The aim of NeoOBS was to evaluate the current clinical treatment practice and the risk factors for poor outcomes of young infants with significant sepsis (including culture-negative, culture-positive, and culture-positive with multi-drug resistant (MDR) pathogens). The primary objective was to estimate 28-day mortality rates in hospitalised infants less than 60 days of age who were being treated for significant sepsis.

The study was designed to inform the design of a global hospital-based trial of novel antibiotic regimens for the management of neonatal sepsis in LMIC settings and settings with highly reported resistance to WHO first-line antibiotic regimens including:

- Data on current antimicrobial treatment of neonates with sepsis and patterns of antimicrobial switching (particularly where the current WHO first-line antibiotics are not being commonly used)
- Data on the clinical presentation of neonates with sepsis to inform trial inclusion criteria (the NeoSep score) and clinical progression (NeoSep Recovery score) [17]
- Understand the differences between sites and the feasibility of collecting high-quality data in different settings
- Identify challenges and solutions to inform the setup and management of the NeoSep1 trial

The aim of this paper is to describe the challenges and pragmatic solutions to managing the NeoOBS study, which can be used to inform future complex multi-centre, multi-country observational studies and pragmatic clinical trials.

## 2. Results

### 2.1. Site Structure

Site study teams differed in size, structure, and research experience introducing complexity in creating and implementing study processes. Some sites had a large, dedicated study team, i.e., study coordinators, research nurses, data entry clerks, and data managers, whereas other teams were coordinated by the principal investigators (PIs) with only a small, dedicated team. Sites were given the flexibility to design their teams in a way that suited their needs and hospital structure within the study budget. Sites with high patient volumes had research teams of approximately 6 to 24 members (average 12 members) including dedicated research fellows and/or nurses, study coordinators, and data clerk/managers. On average, sites had four clinicians, four nurses, one study coordinator, two microbiologists/lab technicians, and one data manager/clerk working on the study. Site teams included routine clinical staff contributing to the study and team members employed directly for the study. Small and/or less research-experienced sites were more vulnerable to the effects of staff changes and national holidays, which had a knock-on effect on data entry timelines and variability in monthly recruitment numbers.

### 2.2. Site Recruitment

Variation in patient volume and case mix was considerable between hospitals, which required site-specific recruitment targets. Sites with high patient volumes (15 sites) had a recruitment maximum of 200 participants, and sites with lower volumes (4 sites) were aiming for 40–100 participants. Sites were able to implement flexible recruitment strategies that reflected the variability in hospital structures (e.g., the ability to screen and recruit potential patients from various neonatal/pediatric departments and provide weekend cover). Sites adopted various strategies for enrolling eligible patients such as displaying study posters/notices in departments with contact details of the research team, which helped non-study clinicians refer potential or readmitted study patients to the appropriate research staff.

Initial site recruitment was capped (two patients for small sites and five patients for large sites) to allow the central study management team (SMT) to conduct 100% remote data monitoring to check eligibility and data quality prior to sites continuing recruitment. Issues such as missing data or outliers were raised and discussed with the site team, and further training was provided when considered necessary. Recruitment processes and data quality were also discussed at this point before recruitment steadily increased.

Sites with high patient volumes and larger research teams were able to sustain a maximum recruitment capacity of 18–20 patients/month while maintaining a high level of data quality. Sites with smaller research teams were able to achieve very good recruitment targets (average 13–15 per month) and sustain high-quality data.

**Figure 1:**
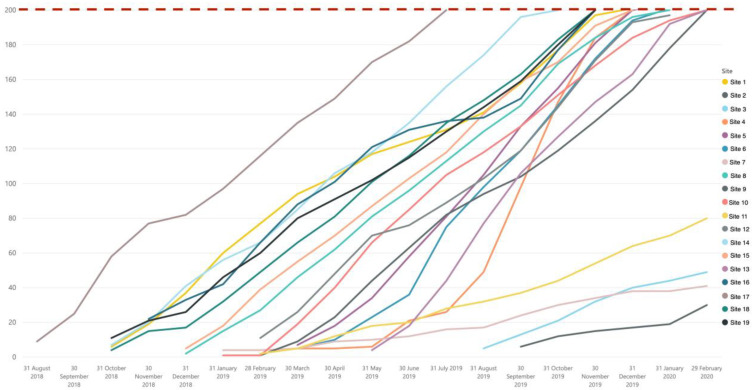
Site-specific recruitment chart for the primary enrolment cohort (clinical sepsis cohort) illustrating 15 high-volume sites meeting the 200-baby target and 4 lower-volume sites recruiting between 35 and 80 babies.

Overall, NeoOBS study successfully recruited 3373 babies through both the primary and secondary recruitment cohorts. The 15 sites with high patient volume were able to meet the recruitment target of 200 babies in the primary enrolment cohort, and the 4 sites with smaller patient volume met expected targets, recruiting 40–80 babies in the primary enrolment cohort (Figure 1) despite delays with ethics and contracts.

### 2.3. Ethics and Regulatory Requirements

**Figure 2:**
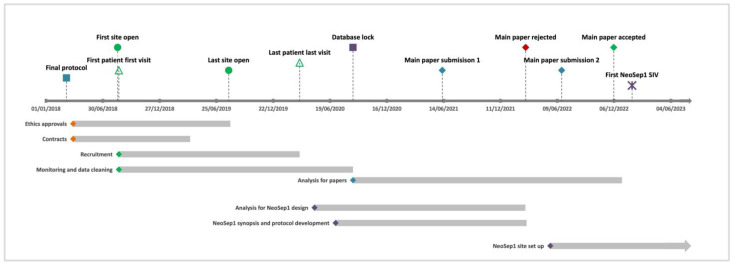
The study timelines for the NeoOBS study include study setup, recruitment, analysis, and the NeoSep1 trial design and setup. The NeoSep1 study setup will continue through 2023.

The master informed consent form (ICF), patient information sheet (PIS), and contract agreement were used by 17 sites, whereas 2 sites used their own templates. All locally adapted documents were reviewed to ensure accurate study processes were being outlined. Sites with multiple levels of ethics approval (country, local/regional, university, hospital level) were prioritised due to the lengthy process of obtaining all approvals. Some delays encountered in obtaining approvals were due to incorrect versions submitted, canceled committee meetings, and length of wait for committee feedback. Research approvals at each site took on average 3.5 months (range 1–8 months), with the average time taken for hospital-level research review and feedback 28 days (range 7–90 days). The time for the site’s contract agreements (CAs) to be signed by all parties took an average of 4.4 months (range: 2–7 months). In total, approximately 18 months were required to open all sites to recruitment (19 sites across 11 countries), although 68% of the sites were open in under 7 months. The overall duration of the study was 30 months from protocol finalisation (March 2018) to database lock (August 2020) (Figure 2).

### 2.4. Study Documents

While the study was primarily conducted in English, patient-facing and study management documents were translated where necessary to ensure accurate information was communicated across the global NeoOBS network (i.e., from sponsor to site principal investigator to parents). Study documents including the protocol, ICF and PIS were translated by professional translators (eight countries), the site (two countries), or the sponsor’s country monitor (one country); this was deemed to be both cost and time effective. Translations of an abbreviated version of the manual of operations and procedures (MOP) and full contracts were professionally translated for sites, where required. Overall, study documents were translated into over 20 languages. The site hospital teams agreed to communicate and collect the study data in English as much as possible, and therefore the case report forms (CRFs), study logs, and REDCap™ database were not translated.

### 2.5. Training and Procedures

The NeoOBS study had a multi-faceted and ongoing training strategy to maintain partnerships with the sites and ensure data quality remained high during the study period.

The site initiation visit (SIV) included visiting the site’s relevant clinical and laboratory departments, where potential bottlenecks were identified and discussed with the site team prior to starting the study; all sites received in-person SIVs. If SIVs were conducted and there were any delays with starting recruitment, remote refresher sessions were offered to re-review study processes before starting recruitment. This also allowed any staff who had joined or had missed the previous session to receive the full SIV.

The NeoOBS study also provided an online training program for the study through The Global Health Network (TGHN) online platform (https://tghn.org/) (accessed on 20 January 2023) [18]. Team members could work through training modules with quizzes on the protocol, enrolment procedures, inclusion criteria, microbiology processes, and blood culture sampling techniques (to minimize contamination). All team members could receive certificates and credits for completing the different modules. As part of this collaboration, all training on TGHN was accessible to sites where they could register, free of charge, to a Professional Development Scheme. This offered each researcher the possibility of getting additional training in different areas of global health and record, tracking and benchmarking their professional development through competencies, qualifications, and training.

Some site teams locally adapted the study manuals to create internal standard operating procedures (SOPs) and implemented internal site study training to ensure all staff worked consistently within their own team. The central SMT had a clear process for reviewing these study documents with site teams, before implementation, to ensure all study processes were consistent at each site and any amendments were reviewed, tracked, approved, and monitored.

Additional training was provided by the central SMT when sites had new members of the team, wishing to have a refresher session, or specific findings during monitoring prompted additional guidance. This increased the work intensity for the central SMT but was regarded as essential to ensure all site processes were conducted correctly.

Regular communications with sites were essential throughout the study. The central SMT conducted monthly calls with each site including both the clinical and microbiology teams from sites to further foster collaboration for the duration of the study. By having a dedicated, easily accessible central SMT, and a clear mode of communication, site teams could send emails or request calls and have queries answered and discussions scheduled quickly. These communications and central SMT structure allowed for successful partnerships to be fostered with sites and provided opportunities for building research skills and experience for all site teams not just the PIs.

### 2.6. Clinical Sepsis Definition and Enrolment Criteria

The variability in clinical severity of neonates with sepsis presenting at sites also introduced challenges when applying the eligibility criteria across all sites. To minimise enrolment difficulties, clinical definitions of signs and symptoms of sepsis were outlined in the study operational manual and were discussed during the site initiation visit and during online training sessions to ensure consistency in recruitment across sites; this also minimised the enrolment of ineligible patients with other conditions such as respiratory distress syndrome. Initially, the inclusion criteria specified the blood culture had to be taken before antibiotic treatment was started; however, at the Investigator Meeting, site teams flagged that many of their babies were already receiving antibiotic treatment or prophylaxis. Based on this feedback, the inclusion criteria were updated to specify the culture taken before new antibiotics were started or there was a change in antibiotic treatment. A pragmatic approach was taken for this criteria as we knew it was not always feasible in clinical practice; sites were encouraged to ensure all staff knew the criteria so culture being taken after antibiotic therapy was initiated could be minimized; however, this was not always possible, especially in very sick patients.

### 2.7. Data Collection Challenges

REDCap™ proved to be an affordable, user-friendly, and a globally accessible system. It was essential to pilot the CRFs and the REDCap™ database with team members across different settings and amend to ensure consistent interpretation of data points.

The number of data points and time taken for data collection and entry into REDCap™ varied widely between patients and sites. Detailed daily prospective data collection was challenging as it required the site team to be very proactive in gathering data from different departments to complete the CRFs. The extensive daily data collection and data entry into the electronic REDCap™ database were a time-burden for all sites, especially for sites with complex patients who remained in the hospital for the whole 28-day study period and were intensively clinically monitored, had multiple changes in treatment and use of devices, and had multiple laboratory and microbiology testing—a patient like this would have had more than 700 data points collected. Site teams had to balance the number of newly enrolled patients with the number of patients in active follow-up to allow enough time for all data to be gathered and entered into the database, to perform data quality checks (e.g., responding to data queries raised during monitoring and conduct site’s own data quality control processes), and to follow-up discharged patients on day 28 either by phone, clinic review appointments, or home visits.

In some instances, some data were not routinely documented in the medical notes [e.g., temperatures within normal range], or specific monitoring (e.g., capillary refill time (CRT)) was not conducted as needed for data collection. In these cases, site study teams were supported by the central SMT to implement study-specific follow-up to ensure these data were captured. Sites used different strategies such as providing additional training to the wider clinical team to record all vital signs whether normal or abnormal in medical notes or ensuring research staff were present during the day and night shifts. Sites had more challenges collecting these data from around the time of blood culture as patients were not always admitted into a department or seen by a clinician who was aware of the study processes, or they were admitted out of hours, or vital signs recordings were often missed or recorded late in the medical notes and CRFs. Through regular monitoring and discussions with sites, central SMT was able to identify these gaps in data collection and work with the site team to develop solutions.

### 2.8. Offsite/Remote Monitoring

The central SMT was able to remotely monitor patient enrolment in near real-time by reviewing anonymized enrolment logs and data in the database to address any issues at the start and throughout the course of the study. The data query audit module in REDCap™ was user-friendly and appropriate for specific data discrepancies to be highlighted and resolved effectively. Many queries raised in REDCap™ were associated with the accuracy and completeness of CRFs, which heavily relied on a dedicated site team effort to ensure there was no missing data or inconsistencies. Over 18,000 data queries were raised in REDCap™ during the study, which was largely due to missing data, inaccurate dates, and extreme data values. Therefore, a large proportion of queries were not data errors, but data checks that were carried out to ensure data was accurate.

To ensure all bacterial isolates were collected from blood and CSF samples, study-specific isolate logs were used to keep track of all isolates, including the identification of the species, storage location, and shipment details. These logs were monitored regularly to ensure all data were reported to the clinical team and matched the data entered by the clinical team into REDCap™ database. This allowed the identification of errors in data entry as well as isolates that were recorded based on microbiology reports but may not have been stored. Early detection of discrepancies and implementation of corrective action(s), due to the intense monitoring by the central SMT, ensured that routine microbiology data collection and isolate storage were conducted according to study procedures and remained consistent throughout the study.

The most common types of missing or unknown retrospective data were maternal factors (e.g., maternal steroids in 7 days prior to delivery and the number of antenatal care visits and intrapartum fever and antimicrobials) with approximately 16% unknown/not reported (range: 10.9% for the intrapartum material fever to 21.7% for maternal receipt of antimicrobials in the 7 days prior to labour. Maternal data were unknown/not reported more commonly in infants more than 3 days postnatal age. Obstetric notes relating to the patient’s mother were often not available due to the baby being born at a different hospital, in the community, or at home, with a lack of access to maternal records (e.g., if the baby was transferred to a specialist Children’s hospital). Therefore, missing/ unknown data were expected for these variables, and it was difficult to resolve.

Off-site monitoring was an efficient, pragmatic process that allowed discrepancies to be detected and corrected early in real-time, reduce the number and cost of on-site visits, and did not jeopardise data protection or data quality. The study had planned two on-site monitoring visits with each site in addition to regular remote monitoring; however, due to the COVID-19 pandemic, on-site monitoring towards the end of the study was challenging due to strict travel restrictions. A total of 16 sites had at least one on-site monitoring visit, and the remaining 3 sites had additional remote monitoring and telephone conferences to compensate for not having a site visit. No difference in data quality was found between sites that had onsite monitoring visits vs. those that had monitoring visits conducted entirely remotely. Regular data checks, data verification against scanned paper CRFs, and regular contact through standing monthly calls with sites, near-daily emails, and ad-hoc video conferences as needed, allowing any issues to be identified early and to work with sites to quickly find solutions.

### 2.9. Microbiology Processes

By the end of the study recruitment (March 2020), all sites [not in China or India] had an agreed material transfer agreement (MTA) in place, and isolates were shipped to the Laboratory of Medical Microbiology at the University of Antwerp, the study central laboratory. Isolates from sites in China and India were not shipped to the central laboratory at UA and were, instead, analysed by a central laboratory within their respective countries. The MTA process took on average 6 months (range: 4–8 months) to complete. Isolates collected from sites in China and India were not exported outside of the country, and therefore arrangements were made with the sponsor and local laboratories within the country to conduct these further analyses in collaboration with the central laboratory at UA. The requirements for exporting isolates varied considerably between sites due to different levels of checks and restrictions (e.g., the need for ethics approval and documentation for airport customs). Overall, most sites were able to ship isolates with no major issues, but some experienced delays due to the COVID-19 pandemic.

The number of isolates collected by each site differed considerably due to variations in the number of patients enrolled and routine practices in repeat cultures being taken and culture-positivity rates. Identification and storage of isolates of study participants required close communication between the clinical study teams and the laboratory teams to ensure the laboratory team knew which patients were enrolled in the study and which isolates to store. A total of 1051 isolates from 13 sites were shipped to the UA for further analyses, including confirmation of identification and molecular characterization by whole-genome sequencing (WGS). A total of 723 isolates belonging to seven distinct pathogens and 21 distinct species were analysed by WGS at the UA central lab.

Overall, sites were able to receive and complete the external quality assurance (EQA) panel from the central laboratory. There were a few delays in shipments due to some sites requiring an import license and administrative hold-ups in airport customs to release the shipment but in general, the process was feasible, and no major issues occurred. Sites identified between 95% (19/20) and 100% (20/20) Gram-negative strains correctly and between 79% (14/19) and 100% (19/19) of carbapenem susceptibility of strains correctly. One site misplaced the EQA panel due to the site’s restructuring of the laboratory and therefore did not complete the testing of the panel. To prevent this issue from occurring in future, it is vital for the central SMT to discuss any potential restructuring of teams or departments that may have an impact on the conduct of the study and ensure that the responsibility for shipments and tasks are given to a dedicated person(s) to carry out.

## 3. Discussion

The NeoOBS study was designed to capture detailed, longitudinal clinical data on the presentation, treatment, management, and outcomes of hospitalised neonates with sepsis in countries with high burdens of antimicrobial resistance. We have described here the challenges and pragmatic solutions of running a complex, longitudinal observational cohort study in 19 hospitals in 11 countries across Africa, Asia, Europe, and South America. The key findings and recommendations are summarised in Table 1.

The NeoOBS study successfully recruited 3373 babies through the primary and secondary recruitment cohorts. All sites with high patient volume (15 sites) reached the target sample size of 200 babies in the primary recruitment cohort. Sites were supported by the central SMT to implement practical recruitment strategies adapted to their local setting and patient volume to achieve the enrolment target.

The main challenge of the study, which is not often reported in study papers, was the complex regulatory and ethics landscape when setting up the global clinical study. It took approximately 18 months from the main protocol being finalised to the last site having all contracts and ethics requirements in place to open. The different templates, translations, and levels of approvals needed varied between sites and countries, which required several rounds of revisions and close coordination with the site partners. This study was an observational study collecting data on routine clinical presentation and management, and more complex studies or interventional studies should account for the complexities and true timelines of approvals when planning new studies.

The study employed comprehensive, multi-modal training and offsite monitoring setup with regular communications with sites and detailed, continuous feedback. Sites were able to achieve high levels of data completeness, with very little missing data for key clinical and microbiological variables. While this was time intensive for sites to balance recruitment and follow up data collection, all sites, regardless of structure and staff numbers, were able to achieve recruitment targets and high-quality data.

The success of the NeoOBS study highlights the feasibility of building highly collaborative research partnerships with global partners to effectively run high-quality large neonatal sepsis studies. Ensuring that central study management processes are not overly prescriptive is important to allow site teams to implement the study protocol and processes in a way that suits local site needs. Close collaboration between site teams and the central study management team enabled the study to be set up effectively in each site, ensuring that local requirements such as ethics, translations, and recruitment strategies were met.

With high rates of antimicrobial resistance reported to the WHO first and second-line empiric antibiotic treatment for neonatal sepsis [8,12,14,19,20], clinical trials exploring novel empiric antibiotic treatment regimens are needed [21,22]. The NeoOBS study aimed to recruit patients who would be recruited in an antibiotic treatment trial and captured high-quality clinical data highlighting the feasibility of running complex studies and trials in partnership with site investigators in LMIC settings. The NeoOBS study partners had a clear common goal, which was to inform the design of the NeoSep1 trial (ISRCTN 48721236) [23], which will compare novel antibiotic combinations [24,25] to the WHO-recommended and other commonly used regimens as reported in the NeoOBS study, with the NeoSep Severity Score guiding trial entry criteria based on the NeoOBS data [17].

## 4. Materials and Methods

### 4.1. Site Structure and Set Up

The NeoOBS hospital partner sites were selected based on a previous feasibility study [7] where information on the size of hospital/units, admission rates, access to supportive therapies, empirical antimicrobial guidelines, and period prevalence of neonatal blood culture isolates and their resistance patterns were collected. The hospitals selected were a mixture of district and tertiary hospitals with varied external research support ranging from non-academic to academic hospital sites. Sites were predominantly tertiary facilities. All sites were required to have high-level local microbiology laboratory services to be eligible for the study. Sites were selected based on the patient burden (neonates with severe sepsis) and the ability of the sites to collect the required data and potentially participate in a future clinical trial.

Prior to the study setup, information regarding the ethical and contractual agreement (CA) processes, costs, approval timelines, and potential issues were gathered from the feasibility survey and site assessments. All sites were provided with master study document templates for parent information sheets (PIS), consent forms (ICF), and contract agreements.

In July 2018, the NeoOBS Investigator Meeting was held in New Delhi, India, to launch and discuss how the study would be implemented with all collaborators and to ensure consistency across all sites. All sites were subsequently visited by the central study management team (SMT) to provide further support and training. The site initiation visits (SIVs) (and previous site feasibility visits) allowed the site and central SMT to help sites to implement the study.

The study operational manuals and associated documents (i.e., parent information sheets (PIS) and consent forms (ICF), case report forms (CRFs), and study logs) were created by the central SMT to ensure consistency of recruitment and data collection between sites. All sites received study-specific training from the central SMT on the processes for recruitment, data collection, query resolution, and microbiology procedures.

### 4.2. Data Collection and Monitoring

The study used REDCap™ [26,27], a secure web-based data collection platform hosted at St. George’s, the University of London, for electronic data capture. Training in data collection, data entry, and query resolution was given to all sites prior to starting recruitment, and regular technical support was provided throughout the study to ensure high data quality. The NeoOBS study collected extensive clinical and antimicrobial treatment data daily plus all routine laboratory and microbiology sampling for the duration of hospitalisation up to 28 days post-enrolment. The number of data points collected for each patient depended on the complexity of the baby (i.e., the number of signs and symptoms of sepsis detected, the number of interventions undertaken, and the number of in-patient days). Vital signs were expected to be recorded prior to/or at the time of the initial enrolment blood culture being taken and to be collected regularly throughout the day (ideally every 4–6 h over a 24-h period).

Offsite monitoring was conducted using the data query audit module in REDCap™, with a detailed review of anonymized electronic copies of the CRFs for the first five patients (for larger sites) and a randomly selected 10% of patients enrolled throughout the study plus routine queries related to missing data and inconsistencies in data entry. Data queries and source data verification were able to be conducted remotely near-real-time, which enabled any issues to be rapidly identified and corrective actions/training implemented. To aid accurate data entry, REDCap™ was designed with data validation (e.g., minimum and maximum cut-offs for weights) and alerts to ensure correct sections and forms were completed. On-site monitoring was conducted by eight independent monitors selected by the sponsor (several through the sponsor’s global partner offices) to verify data, highlight issues with data entry, and provide re-training if required; on-site monitoring was structured to be conducted once per site during the study.

### 4.3. Microbiology Processes

As part of the study, bacterial isolates from blood and CSF cultures of participating babies were collected at all sites and stored at a minimum of −20 °C (ideally −70 °C where a freezer was available) per the study laboratory operational manual. All sites except those in China and India shipped isolates to the central laboratory at the Laboratory of Medical Microbiology at the University of Antwerp (UA) at the end of the study for study-specific analyses. All sites were given training on how to collect and store the isolates. Amendments were made to the PIS and ICF for all sites excluding those in China and India to inform parents that bacterial isolates collected from their baby’s blood or CSF culture(s) would be collected, stored, and exported to the central laboratory at UA for further analyses. All isolates were stored in duplicates in 2 mL microbanks at −80 °C at the local laboratories until shipment to the central laboratory at the University of Antwerp. One set of the collected isolates remained stored locally as a backup until re-identification at the central laboratory. Material transfer agreement (MTA) templates had to be adapted for observational studies to facilitate approvals in all countries. Some sites used their own national template, which led to additional discussions and delays in signing final agreements.

Most sites completed an External Quality Assurance (EQA) microbiology panel with the aim of objectively evaluating laboratory capacity to detect and accurately identify multi-drug resistant (MDR) Gram-negative pathogens and test their antimicrobial susceptibility during the NeoOBS Study. An EQA panel, which consisted of 20 investigator-blinded Gram-negative strains, was sent to the microbiology teams at 16 hospital sites between June and August 2019, for isolate identification and detection of resistance to carbapenems using routine phenotypic methods. EQA panels were not sent to sites in China due to import–export restrictions. The strains were selected from the biorepository of the central laboratory and were well-characterised for identification to the species level using MALDI-TOF, as well as for susceptibility, and resistance phenotypes using disc diffusion, broth microdilution, and PCR targeting selected resistance genes. This panel included multidrug-resistant Enterobacterales strains with various resistance mechanisms to carbapenems (mostly carbapenemase producers belonging to the major family types) or resistance to broad-spectrum beta-lactams (3rd and/or 4th generation cephalosporins, and penicillins combined with beta-lactamase inhibitors) through the production of extended-spectrum beta-lactamases (ESBL) and/or AmpC cephalosporinases. Sites entered the results into an EQA-panel-specific web-based IT tool that had been developed by the central laboratory together with the data management team at the Universitair Medisch Centrum Utrecht (UMCU).

## 5. Conclusions

The success of the NeoOBS study in collecting detailed, high-quality, longitudinal clinical and microbiological data in 19 hospitals in 11 countries globally highlights the feasibility of building completely new collaborative global networks for complex clinical studies focused on a common vision. The creative and adaptable study management approach utilised in NeoOBS ensured the study was locally implementable but consistent across sites. The challenges and solutions used within this study may be useful to guide strategies for future neonatal clinical trials.

## Figures and Tables

**Table 1:** Key recommendations and solutions for global pragmatic studies as found from the NeoOBS study experience.

Key Recommendations	Successful Strategies and Considerations
1 It is possible to do complex observational studies in diverse hospital sites	Central study teams and protocols should take a pragmatic approach and allow for the adoption of site-specific processes/solutions to be implemented.
2 Regular communication and building partnership with sites are essential for the success of the project	Having multi-modal regular communications with all members of the site teams (PIs, microbiologists, data managers, research nurses, etc.) allowed for issues to be identified and addressed in a timely manner. Engaging all members of the study team meant solutions could be designed with those doing much of the day-to-day study work and tailored to each site.
3 Regular training in multiple formats ensured continuous feedback and good adherence to study processes	Live video training, written resources, and online modules [on TGHN] as well as site-specific training needed during the study (e.g., on specific findings from monitoring) allowed for ongoing engagement and high-quality study conduct/data. It also provided an opportunity for site team members to build skills for their own roles that may potentially further benefit patient care and their research careers.
4 Offsite, remote monitoring was highly efficient, cost-effective and delivered high data quality	A combination of data queries, data verification with paper CRFs, and building data checks automatically into the database delivered high data quality and allowed for detailed feedback and discussion with sites on an ongoing basis. Given the high costs and the carbon footprint of onsite monitoring visits for global studies, the NeoOBS study demonstrated that data quality is not necessarily compromised with an offsite monitoring approach.
5 Ethics and contracts take a considerable amount of time and different country/site requirements and regulations need to be considered	The realistic study setup timelines need to be considered by sponsors and funders, allowing sufficient time at the beginning of the study to discuss site/country-specific issues, and cultural differences will lead the way to a successful study.

## Data Availability

The individual participant data, after deidentification may be shared on reasonable request. Researchers who wish to use the data for further analyses should submit a proposal to GARDP (study sponsor). This will be reviewed by the investigators, and collaborators for approval, and depend on a data sharing agreement.

## Appendix A

**Table A1:** Detailed inclusion criteria for the NeoOBS study for both the clinical sepsis cohort (primary enrolment cohort) and the microbiology cohort (a secondary enrolment cohort).

	Clinical Sepsis Cohort (Primary Enrolment Cohort)	Microbiology Cohort (Secondary Enrolment Cohort)
**Inclusion criteria**	In-patient in the hospital of this institution (neonatal unit, paediatric ward, or emergency department) Age <60 days (postnatal age) Informed consent from parent/guardian Parent provided contact information for 28-day follow-up Clinical suspicion of a new episode of sepsis (defined below*) together with planned treatment with IV antibiotics	In-patient in the hospital of this institution (neonatal unit, paediatric ward, or emergency department) Age <60 days (postnatal age) Informed consent from parent/guardian Parent provided contact information for 28-day follow-up One of the following microbiology findings: ○ New episode of infection in which a Candida species is isolated from blood culture OR ○ New episode of infection in which a carbapenem-resistant organism (CRO) is isolated from blood culture OR ○ New episode of confirmed bacterial meningitis—defined as (A) or (B) below ▪ Isolation of a significant bacterial pathogen from cerebrospinal fluid (CSF) ▪ Isolation of a significant bacterial pathogen from blood cultures AND CSF white cells ≥20 cells/mm^3^ (for babies 0–28 days of age) or CSF white cells ≥ 10 cells/mm^3^ (for babies 29–60 days of age)
**Exclusion Criteria**	Previously enrolled in this study Enrolment in any interventional trial A serious, non-infective co-morbidity (other than prematurity) that is anticipated to cause death within 72 h	
*** Definition of clinical sepsis**	Baby must have at least two clinical/laboratory signs of which at least one must be a clinical sign. Second criteria may be either clinical or laboratory sign. Clinical Criteria Capillary refill time (CRT) >3 s or mottled skin or other evidence of shock Multiple or severe skin pustules Petechial rash Pus from umbilical stump Severe chest in-drawing or increased oxygen requirement or need for respiratory support Grunting Apnoea Cyanosis Abnormal heart rate (>180/min or <100/min) Abnormal temperature (>37.5 °C or <36.5 °C) or temperature instability (i.e., wide variations) Irritability Convulsions Abnormal posturing Hypotonia/floppiness Lethargy or drowsiness Bulging fontanelle No movement or movement only when stimulated Difficulty feeding or feeding intolerance Abdominal distension Laboratory Criteria White blood cells (WBC) count < 4 × 109 cells/L or >20 × 109 cells/L Absolute neutrophil count < 1.5 × 109 cells/L Immature-to-total (ITT) polymorph ratio > 0.2 C-reactive protein (CRP) >10 mg/L or >1 mg/dL Acidosis: Base excess < −10 mmol/L or blood lactate >2 mmol/L	

## References

[1] Thaver D., Ali S.A., Zaidi A.K.M. (2009). Antimicrobial resistance among neonatal pathogens in developing countries. Pediatr. Infect. Dis. J..

[2] Morkel G., Bekker A., Marais B.J., Kirsten G., van Wyk J., Dramowski A. (2014). Bloodstream infections and antimicrobial resistance patterns in a South African neonatal intensive care unit. Paediatr. Int. Child Health.

[3] Jajoo M., Manchanda V., Chaurasia S., Sankar M.J., Gautam H., Agarwal R., Yadav C.P., Aggarwal K.C., Chellani H., Ramji S. (2018). Alarming rates of antimicrobial resistance and fungal sepsis in outborn neonates in North India. PLoS ONE.

[4] Reddy K., Bekker A., Whitelaw A.C., Esterhuizen T.M., Dramowski A. (2021). A retrospective analysis of pathogen profile, antimicrobial resistance and mortality in neonatal hospital-acquired bloodstream infections from 2009–2018 at Tygerberg Hospital, South Africa. Duse AG, editor. PLoS ONE.

[5] Chaurasia S., Sivanandan S., Agarwal R., Ellis S., Sharland M., Sankar M.J. (2019). Neonatal sepsis in South Asia: Huge burden and spiralling antimicrobial resistance. BMJ.

[6] Le Doare K., Bielicki J., Heath P.T., Sharland M. (2015). Systematic review of antibiotic resistance rates among gram-negative bacteria in children with sepsis in resource-limited Countries. J. Pediatric. Infect. Dis. Soc..

[7] Li G., Bielicki J.A., Ahmed A.N.U., Islam M.S., Berezin E.N., Gallacci C.B. (2020). Towards understanding global patterns of antimicrobial use and resistance in neonatal sepsis: Insights from the NeoAMR network. Arch. Dis. Child.

[8] Folgori L., Bielicki J. (2019). Future Challenges in Pediatric and Neonatal Sepsis: Emerging Pathogens and Antimicrobial Resistance. J Pediatr. Intensive Care.

[9] UN Inter-Agency Group for Child Mortality Estimation (2019). Levels and Trends in Child Mortalit.

[10] SDG Goal 3: Good Health and Well-Being-UNICEF DATA. https://data.unicef.org/sdgs/goal-3-good-health-wellbeing/#cme_mry0t4.

[11] Fleischmann-Struzek C., Goldfarb D.M., Schlattmann P., Schlapbach L.J., Reinhart K., Kissoon N. (2018). The Global Burden of Paediatric and Neonatal Sepsis: A Systematic Review.

[12] Investigators of the Delhi Neonatal Infection Study (DeNIS) collaboration (2016). Characterisation and antimicrobial resistance of sepsis pathogens in neonates born in tertiary care centres in Delhi, India: A cohort study. Lancet Glob. Health.

[13] Sands K., Carvalho M.J., Portal E., Thomson K., Dyer C., Akpulu C., Walsh T.R. (2021). Characterization of antimicrobial-resistant Gram-negative bacteria that cause neonatal sepsis in seven low- and middle-income countries. Nat. Microbiol..

[14] Thomson K.M., Dyer C., Liu F., Sands K., Portal E., Carvalho M.J., Barrell M., Boostrom I., Dunachie S., Farzana R. (2021). Effects of antibiotic resistance, drug target attainment, bacterial pathogenicity and virulence, and antibiotic access and affordability on outcomes in neonatal sepsis: An international microbiology and drug evaluation prospective substudy (BARNARDS). Lancet Infect. Dis..

[15] Saha S.K., Schrag S.J., el Arifeen S., Mullany L.C., Shahidul Islam M., Shang N., Qazi S.A., Zaidi A.K.M., Bhutta A.Z., Bhutta A. (2018). Causes and incidence of community-acquired serious infections among young children in south Asia (ANISA): An observational cohort study. Lancet.

[16] Russell N., Stöhr W., Plakkal N., Cook A., Berkley J.A., Adhisivam B., Agarwal R., Balasegaram M., Ballot D., Bekker A. (2022). Patterns of antibiotic use, pathogens and clinical outcomes in hospitalised neonates and young infants with sepsis in the NeoOBS global neonatal sepsis observational cohort study. medRxiv.

[17] Russell N., Stöhr W., Cook A., Berkley J.A., Adhisivam B., Agarwal R., Ahmed N.U., Balasegaram M., Chami N., Bekker A. (2022). The NeoSep Severity and Recovery scores to predict mortality in hospitalized neonates and young infants with sepsis derived from the global NeoOBS observational cohort study. medRxiv.

[18] NeoOBS Study Global Health Training Centre. https://globalhealthtrainingcentre.tghn.org/login/?next=/elearning/education/NeoObs_Study_Training.

[19] Wen S.C.H., Ezure Y., Rolley L., Spurling G., Lau C.L., Riaz S., Irwin A.D. (2021). Gram-negative neonatal sepsis in low- and lower-middle-income countries and WHO empirical antibiotic recommendations: A systematic review and meta-analysis. PLoS Med..

[20] Folgori L., Bielicki J., Heath P.T., Sharland M. (2017). Antimicrobial-resistant Gram-negative infections in neonates. Curr. Opin. Infect. Dis..

[21] Williams P.C.M., Qazi S.A., Agarwal R., Velaphi S., Bielicki J.A., Nambiar S., Sharland M. (2022). Antibiotics needed to treat multidrug-resistant infections in neonates. Bull. World Health Organ..

[22] Oeser C., Lutsar I., Metsvaht T., Turner M.A., Heath P.T., Sharland M. (2013). Clinical trials in neonatal sepsis. J. Antimicrob. Chemother..

[23] ISRCTN-ISRCTN48721236: NeoSep1: A Study to Determine the Ranking of Existing and New Antibiotics Combinations to Treat Newborn Babies Who Are in Hospital with Severe Sepsis. https://www.isrctn.com/ISRCTN48721236.

[24] Obiero C.W., Williams P., Murunga S., Thitiri J., Omollo R., Walker A.S., Egondi T., Nyaoke B., Correia E., Kane Z. (2022). Randomised controlled trial of fosfomycin in neonatal sepsis: Pharmacokinetics and safety in relation to sodium overload. Arch. Dis. Child.

[25] Williams P.C.M., Waichungo J., Gordon N.C., Sharland M., Murunga S., Kamau A., Berkley A. (2019). The potential of fosfomycin for multi-drug resistant sepsis: An analysis of in vitro activity against invasive paediatric gram-negative bacteria. J. Med. Microbiol..

[26] Harris P.A., Taylor R., Minor B.L., Elliott V., Fernandez M., O’Neal L., McLeod L., Delacqua G., Delacqua F., Kirby J. (2019). The REDCap consortium: Building an international community of software platform partners. J. Biomed. Inform..

[27] Harris P.A., Taylor R., Thielke R., Payne J., Gonzalez N., Conde J.G. (2009). Research electronic data capture (REDCap)—A metadata-driven methodology and workflow process for providing translational research informatics support. J. Biomed. Inform..

